# Immune-Related Adverse Events in Breast Cancer Patients Who Received Neoadjuvant Chemotherapy with Pembrolizumab: What Needs to Be Managed Before Surgery

**DOI:** 10.3390/cancers18060919

**Published:** 2026-03-12

**Authors:** Jeeyeon Lee, Byeongju Kang, Joon Suk Moon, Taegyu Um, Jung Eun Choi, Moohyun Lee, Yee Soo Chae, Soo Jung Lee, In Hee Lee, Soo Jung Lee, Su Hwan Kang, Sung Ae Koh, Sun Hee Kang, Keon Uk Park, Hyera Kim, Ho Yong Park

**Affiliations:** 1 School of Medicine, Kyungpook National University, Kyungpook National University Chilgok Hospital, Daegu 41404, Republic of Korea; j.lee@knu.ac.kr (J.L.); libertas033@knu.ac.kr (B.K.); joonsukm@knu.ac.kr (J.S.M.); alfred20@naver.com (T.U.); yschae@knu.ac.kr (Y.S.C.); sj.lee@knu.ac.kr (S.J.L.); ihleeoncology@knu.ac.kr (I.H.L.); 2 College of Medicine, Yeungnam University, Daegu 42415, Republic of Korea; cjegs@yu.ac.kr (J.E.C.); crystallee@med.yu.ac.kr (S.J.L.); kangsuhwan@yu.ac.kr (S.H.K.); sakoh@yu.ac.kr (S.A.K.); 3 School of Medicine, Keimyung University, Daegu 42601, Republic of Korea; mhlee197@dsmc.or.kr (M.L.); shkang9002@dsmc.or.kr (S.H.K.); kupark@dsmc.or.kr (K.U.P.); kheyra@hanmail.net (H.K.)

**Keywords:** breast cancer, neoadjuvant chemotherapy, immunotherapy, irAEs, surgery

## Abstract

**Background:** Pembrolizumab is a novel immunotherapy agent that improves oncological outcomes for various cancers. This study aimed to investigate immune-related adverse events (irAEs) that occurred after neoadjuvant chemotherapy (NAC) with pembrolizumab for triple-negative breast cancer (TNBC) and to identify fatal irAEs that should be addressed before surgery under general anesthesia. **Methods:** A total of 82 patients who received NAC with pembrolizumab followed by surgery were reviewed based on their medical records. All irAEs during NAC were evaluated and classified into nine categories: systemic, dermatologic, central nervous, musculoskeletal, endocrine, gastrointestinal, respiratory, ocular, and hematologic systems. Clinicopathologic characteristics were compared between patients with and without irAEs, and cases in which surgery was postponed due to irAEs were reviewed and analyzed. **Results:** Fifty-nine patients (72.0%) experienced irAEs after NAC with pembrolizumab. The mean NAC period was 140.3 days, with a 39.8-day window to surgery. The most common and second most common irAEs were myalgia (n = 33, 40.3%) and skin rash/dermatitis (n = 31, 37.8%), respectively. There were 1 case (1.2%) of adrenal insufficiency and 16 cases (19.5%) of thyroid dysfunction as irAEs. In 6 cases (7.3%), surgery was postponed due to irAEs [systemic (n = 2, 33.3%); endocrine (n = 3, 50.0%); increased transaminase (n = 1, 16.7%)], with a mean delay of 64.5 days (range, 57–80 days). **Conclusions:** IrAEs following NAC with pembrolizumab in TNBC were diverse and included those severe enough to affect the timing of surgery under general anesthesia. These irAEs should be monitored continuously during NAC and detected early to address them when they occur.

## Simple Summary

Pembrolizumab combined with chemotherapy is increasingly used before surgery in triple-negative breast cancer, but it can cause immune-related side effects. In this study, most adverse events were mild, yet some—such as thyroid dysfunction and liver toxicity—delayed surgery. Careful monitoring during treatment is essential to prevent unnecessary postponement and ensure safe surgical care.

## 1. Introduction

Although immunotherapy for the treatment of cancers has been proposed for more than a century [1,2,3], breast cancer has been excluded because it has not been considered immunogenic. However, advances in immune checkpoint inhibitors (ICIs) have led to the improvement of oncologic outcomes in triple-negative breast cancer (TNBC) [4,5]. In particular, pembrolizumab (Keytruda^®^; Merck Sharp & Dohme, London, UK), an anti–programmed death 1 (PD-1) monoclonal antibody, has been reported to have relatively low toxicity and contribute to improved survival rates in patients with metastatic TNBC [6,7]. Furthermore, this treatment strategy could be applied to patients with advanced, but operable TNBC in neoadjuvant chemotherapy (NAC) to achieve a pathologic complete response (pCR) [8,9].

As pembrolizumab binds to PD-1 without engaging Fc receptors or activating complements, the cytotoxic effect is low [10]. However, treatment using ICIs can cause various immune-related adverse events (irAEs) by disrupting the balance of the immune system [11,12]. Although most irAEs are manageable, several irAEs can be fatal or delay additional treatments, including surgery. In particular, if irAEs in the respiratory system (e.g., pneumonia or pneumonitis) or endocrine system (e.g., adrenal insufficiency, hyperthyroidism, or hypothyroidism) have occurred, surgical postponement becomes inevitable, and the treatment effect on the disease will be reduced.

As immunotherapy for breast cancer is often used in combination with chemotherapeutic agents, it is difficult to determine whether irAEs are attributed to chemotherapy or immunotherapy. In addition, the severity of irAEs can vary, and they may even exacerbate each other [13,14]. Unlike situations where immunotherapy is administered as a standalone treatment, such as for non-small cell lung cancer (NSCLC) or renal cell carcinoma (RCC), a combination approach in breast cancer requires a more nuanced evaluation [15,16,17]. However, most pivotal trials have primarily focused on oncologic efficacy and overall safety profiles, while limited data are available regarding the perioperative implications of irAEs in operable TNBC. In particular, the impact of irAEs on surgical timing, perioperative management, and treatment continuity in real-world settings has not been sufficiently characterized. Furthermore, few studies have systematically evaluated which specific toxicities may necessitate intervention before definitive surgery. Therefore, a more detailed understanding of irAEs in the neoadjuvant setting is clinically relevant for optimizing surgical planning and multidisciplinary coordination.

This study aimed not only to evaluate the incidence and spectrum of irAEs after pembrolizumab-based NAC in TNBC, but also to assess their perioperative impact and identify clinically actionable toxicities that may influence surgical timing.

Therefore, it is important to recognize that adverse events may stem from the combined effects of immunotherapy and chemotherapy, and complications that may be fatal or affect surgery or anesthesia should be evaluated and corrected in advance.

This study evaluated irAEs after NAC with pembrolizumab in patients with TNBC and determined which irAEs should be diagnosed and treated before surgery to prevent any delays in treatment.

## 2. Methods

### 2.1. Study Population

Between January 2022 and May 2024, 82 patients with TNBC who were diagnosed at Kyungpook National University Chilgok Hospital, Yeungnam University Hospital, and Dongsan Hospital of Keimyung University underwent NAC combined with pembrolizumab. When the cancer was diagnosed as stage II-III TNBC according to immunohistochemical staining, the stage of breast cancer was assessed, and the treatment strategy was established. Based on KEYNOTE-522 [8], NAC was conducted with four cycles of pembrolizumab (at a dose of 200 mg) every 3 weeks plus paclitaxel and carboplatin, followed by four cycles of pembrolizumab plus doxorubicin–cyclophosphamide.

Informed consent was waived due to the retrospective study design, and this study was approved by the Institutional Review Board Committee (DGIRB 2024-01-001).

### 2.2. Evaluation of irAEs

Before NAC with pembrolizumab, the underlying diseases and patient condition were evaluated, and it was determined whether immunotherapy could be performed. The irAEs were categorized as systemic, dermatologic, central nervous, musculoskeletal, digestive, respiratory, endocrine, and ocular systems. In addition, the irAEs were classified and graded according to the Common Terminology Criteria for Adverse Events (CTCAE) version 5.0 [18]. In accordance with guidelines published by the Society for the Immunotherapy of Cancer [19], the patients were monitored for irAEs during NAC by symptom assessment, physical examination, and laboratory blood tests, including complete blood count, liver and renal function tests, electrolytes, thyroid function test (TFT), glucose, and lipid profiles. TFT was performed every 4 cycles of NAC, and adrenocorticotropic hormone (ACTH) stimulation test was performed between the completion of NAC and surgery to evaluate adrenal insufficiency. Echocardiography was also performed at baseline and at the completion of NAC or if the patients experienced any cardiac symptoms. The types of irAEs were as follows: systemic system (myalgia, fever); dermatologic system (skin rash/dermatitis, soft tissue swelling/cellulitis, hyperpigmentation, Herpes zoster infection); central nervous system (peripheral neuropathy, headache); musculoskeletal system (arthralgia, bone pain); digestive system (diarrhea, constipation, nausea/vomiting, dyspepsia, anal fissure, elevation of liver enzymes); respiratory system (dyspnea on exertion, pneumonitis, pneumonia, ground-glass opacity, non-tuberculous mycobacteria infection); endocrine system (hypothyroidism, hyperthyroidism, adrenal insufficiency); ocular system (uveitis).

### 2.3. Clinicopathologic Characteristics

The size, number, and location of breast cancer lesions were identified by mammography, ultrasonography, and breast MR imaging prior to treatment, and distant metastasis was evaluated with chest/abdomen CT and bone scan. In this study, the clinical variables assessed were age, body mass index (BMI), underlying diseases (e.g., hypertension and diabetes mellitus), incidence of bilateral breast cancer, type of breast and axillary surgery, rate of breast reconstruction, rate of radiotherapy, period of NAC, and period between the end of NAC and surgery. Disease characteristics were assessed, such as the type of tumor, clinical tumor size, clinical T, N, and overall stage, rate of pCR, pathologic size of invasive carcinoma after NAC, and pathologic T and N stage. Variables were compared between patients with and without irAEs.

### 2.4. Statistical Analyses

Statistical analyses were performed using SPSS version 29.0 (IBM Corporation, Armonk, NY, USA). For the comparison of the irAE group and no irAE group, categorical or continuous variables were evaluated using the Chi-square test or Student’s *t*-test. Statistical significance was defined as *p* < 0.05.

## 3. Results

The mean age of 82 patients was 50.1 (SD, ±10.2) years, and the mean BMI was 23.5 (SD, ±3.0) kg/m^2^. The most frequent underlying diseases of the patients were hypertension (n = 10, 12.2%) and hyperlipidemia (n = 10, 12.2%). In addition, there were several immune-related underlying diseases including rheumatic disease (n = 3, 3.7%), autoimmune hypothyroidism (n = 1, 1.2%), and systemic lupus erythematosus (n = 1, 1.2%). A total of 55 patients (67.1%) received breast-conserving surgery, and 64 patients (78.0%) underwent sentinel lymph node biopsy or axillary sampling. The mean period of NAC was 140.3 (SD, ±31.7) days, and the mean period between the end of NAC and surgery was 39.8 (SD, ±20.1) days. Due to sparse data and quasi-complete separation across stage categories, odds ratios for clinical and pathologic stage variables were not estimable (Table 1).

A total of 59 patients (72.0%) experienced irAEs after NAC with pembrolizumab, and 23 patients (28.0%) did not experience irAEs. The frequencies of irAEs were as follows: 1 (n = 11, 13.4%), 2 (n = 20, 24.4%), and ≥3 (n = 22, 26.8%). There was no statistical difference in age, BMI, bilaterality, or type of breast or axillary surgery. Although breast reconstruction was more frequently performed in the irAE group (n = 14, 23.7%) than in the no irAE group (n = 2, 8.7%), and adjuvant radiotherapy was more frequently performed in the no irAE group (n = 22, 95.7%) than in the irAE group (n = 46, 78.0%), there was no statistical significance (*p* = 0.147, *p* = 090). The period between the end of NAC and surgery was significantly longer in the irAE group than in the no irAE group (*p* = 0.058). There was no statistical difference in the tumor types, rate of pCR after NAC with pembrolizumab and clinical and pathologic stages between the two groups (Table 2).

**Figure 1:**
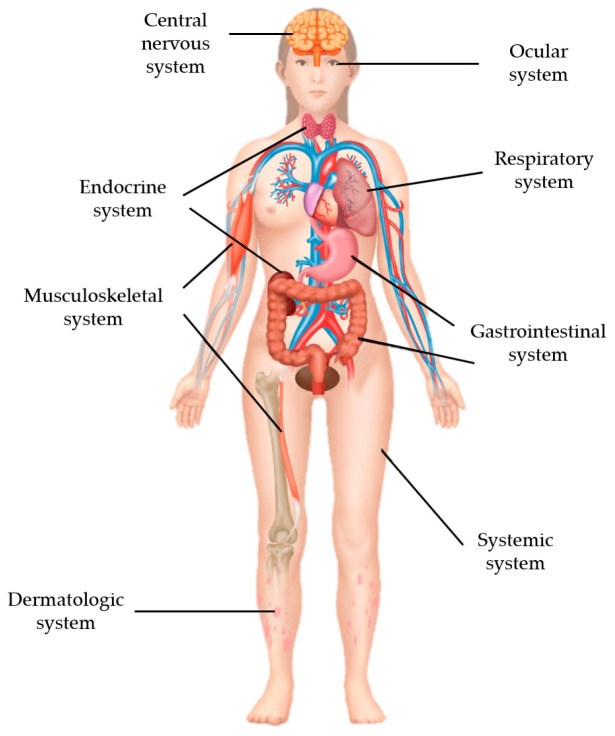
Organ systems of immune-related adverse events after neoadjuvant chemotherapy with pembrolizumab for triple-negative breast cancer.

**Figure 2:**
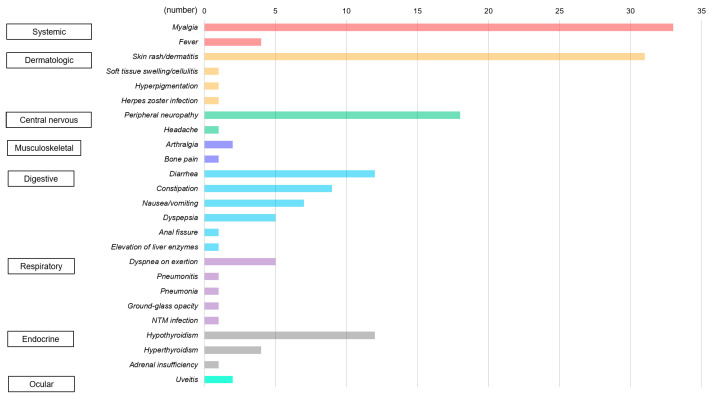
Bar graph showing the types and distribution of immune-related adverse events by organ system after pembrolizumab-based neoadjuvant chemotherapy for triple-negative breast cancer.

There were irAEs after NAC with pembrolizumab in various body systems, including the systemic, dermatologic, central nervous, musculoskeletal, endocrine, digestive, respiratory, and ocular systems (Figure 1). The most common and second most common irAEs were myalgia (n = 33, 40.32%) and skin rash/dermatitis (n = 31, 37.8%). Peripheral neuropathy (n = 18, 22.0%), thyroid dysfunction (hypothyroidism: n = 12, 14.6%; hyperthyroidism: n = 4, 4.9%), and diarrhea (n = 12, 14.6%) were relatively common irAEs after NAC with pembrolizumab. Grade 2 adrenal insufficiency occurred in 1 case (1.2%), which was managed with medical intervention. In the hematologic system, there was 1 case (1.2%) of grade 3 anemia and 1 case (1.2%) of grade 3 increased transaminase (Table 3, Figure 2).

Of the 82 patients, 6 patients (7.3%) required the postponement of surgery after NAC due to irAEs. Patient #33 had fever, mucositis (grade 3), and anemia (grade 3) with general weakness, and patient #35 showed general weakness with poor oral intake despite no abnormalities in laboratory or imaging findings. Patients #38, #48, and #78 developed hypothyroidism after NAC and pembrolizumab, with thyroid-stimulating hormone (TSH) levels greater than 50 mIU/L. They were all replaced with levothyroxine, and surgery was performed under general anesthesia after TSH levels became less than 5 mIU/L. Patient #55 showed increased transaminase (aspartate transaminase, 841 U/L; alanine transferase, 502 U/L) as a grade 3 irAE. The mean period between the end of NAC and surgery was 64.5 days (range, 57–80 days), and 2 patients could not complete the NAC protocol as scheduled. Patient #33 stopped NAC after cycle #5 due to poor general conditions, and patient #55 stopped NAC only after cycle #1 due to a grade 3 irAE, which was increased transaminase (Table 4).

## 4. Discussion

Various irAEs occurred after pembrolizumab-based NAC in patients with TNBC, with the most common irAE being myalgia and the second most common irAE being skin rash/dermatitis. Most of these irAEs were non-fatal; however, several irAEs were graded higher than 2 based on the CTCAE and required intervention or management. Surgery was delayed due to 6 irAEs (grade ≥ 2), half of which were related to hypothyroidism, which led to inadequate general anesthesia. These results suggest that irAEs should be evaluated continuously during NAC. In addition to patient-rated symptoms, factors that must be addressed or may delay surgery, such as abnormal thyroid function and adrenal insufficiency, should be monitored continuously as these irAEs may vary from patient to patient.

TNBC is characterized by an aggressive phenotype and very limited treatment options, leading to high rates of recurrence or metastasis. NAC has been the preferred treatment option for operable stage II or III TNBC [20,21,22]. However, recent results from the KEYNOTE-522 study have significantly expanded the treatment options for TNBC through immunotherapy [4,5,8,9,22]. The addition of pembrolizumab to taxane-platinum followed by anthracycline-based chemotherapy was observed to significantly increase pCR rates and improve event-free survival in early-stage high-risk TNBC [8,9,23]. However, irAEs have not been evaluated and analyzed sufficiently.

Severe irAEs are rare but can sometimes be life-threatening and may delay surgery [24,25]. Therefore, continuous monitoring of irAEs during NAC is crucial to reduce patient mortality and prevent treatment delays. Immunotherapy for NSCLC or RCC is often administered alone [15,16,17], and irAEs in these malignancies have been extensively examined due to the long-term application of immunotherapy [26,27,28,29,30,31,32]. However, in the case of breast cancer, immunotherapy is primarily used in combination with chemotherapeutic agents. As its application in breast cancer is relatively recent, there is still a lack of comprehensive reports on irAEs.

In this study, all irAEs in TNBC patients who received NAC with pembrolizumab were recorded. Several irAEs were graded ≥ 2, requiring management based on the CTCAE. Among cases with irAE requiring management, 6 cases required prolonged treatment, delaying surgery for more than 8 weeks after the end of NAC. Of these cases, 50% involved grade 2 hypothyroidism with TSH abnormalities severe enough to preclude general anesthesia. Thyroid hormones play a crucial role in maintaining homeostasis, affecting the cardiovascular, respiratory, renal, gastrointestinal, hematologic, and central nervous systems. In the cardiovascular system, thyroid hormone imbalances can lead to life-threatening complications, including a risk of coronary events [33], prolonged half-life of multiple coagulation factors [34,35], non-specific ST changes and low voltage on electrocardiograms, and, in rare cases, “torsade de pointes” ventricular tachycardia [36]. Specifically, hypothyroidism is associated with significantly decreased cardiac output, characterized by both a slowed pulse and reduced contractility [37,38,39]. When levothyroxine is taken orally or administered via intravenous injection, it has a half-life of approximately 7.5 days when taken daily [40]. Therefore, a period of around 6 weeks is required for it to reach a steady level in the blood [41]. Considering this correction period, thyroid function should be continuously monitored during NAC to prevent a decrease in therapeutic effect due to surgical delay.

Although hypothyroidism was the most frequently observed irAE in our cohort, it was generally low grade and manageable with hormone replacement therapy, rarely necessitating interruption of systemic treatment. In contrast, less common but clinically more consequential toxicities—such as immune-related pneumonitis and grade 3 transaminase elevation—may exert a disproportionate impact on perioperative planning. Pneumonitis, even when mild at presentation, carries the potential for rapid progression and may require systemic corticosteroid therapy, temporary discontinuation of immunotherapy, or additional radiologic monitoring. Similarly, significant hepatic toxicity can delay subsequent treatment cycles and necessitate close biochemical surveillance until liver function stabilizes.

From a surgical perspective, these toxicities are particularly relevant because unresolved thyroid dysfunction, pulmonary or hepatic dysfunction may increase perioperative risk and influence the optimal timing of definitive surgery. Therefore, early detection through structured laboratory and symptom monitoring, along with timely collaboration between oncologists, surgeons, endocrinologists, and other relevant specialists, is essential. Proactive management strategies may help minimize treatment interruption, avoid unnecessary surgical delay, and ensure that patients safely proceed to curative-intent surgery without compromising oncologic outcomes.

A limitation of this study is the difficulty in distinguishing whether the observed adverse events were related to chemotherapy, pembrolizumab, or their combined effects. However, immune-mediated toxicities such as thyroid dysfunction and adrenal insufficiency have been consistently reported with pembrolizumab in the KEYNOTE-522 trial and are biologically plausible consequences of PD-1 blockade. Therefore, regardless of exact attribution, careful monitoring of these events during neoadjuvant therapy remains clinically important, particularly before surgery. Another limitation of this study is that postoperative outcomes were not evaluated. As the analysis was restricted to the neoadjuvant period, the follow-up duration was limited to approximately six months, and the impact of preoperative irAEs on long-term surgical or oncologic outcomes could not be assessed. Additionally, this study targeted a relatively small number of Asian female patients; thus, the results may differ in a large-scale study or in cases where there are differences in immune responses among races [42,43]. And as a retrospective study, this analysis may be subject to selection bias, limited generalizability, and potential underreporting of mild symptoms, although objective laboratory parameters were routinely monitored. However, regardless of the type of irAE, continuous monitoring and early detection during NAC may help prevent delays in subsequent treatments.

## 5. Conclusions

IrAEs may occur across multiple organ systems during pembrolizumab-based neoadjuvant chemotherapy for TNBC, and some may necessitate active management prior to surgery. Continuous monitoring—particularly of endocrine function and relevant laboratory parameters—is essential during the neoadjuvant period. Early recognition and appropriate management of these events may help prevent surgical delay and optimize overall treatment continuity.

## Figures and Tables

**Table 1:** Clinical characteristics of patients who received pembrolizumab-based neoadjuvant chemotherapy.

Variables	Total (n = 82)	irAE * (n = 59)	No irAE (n = 23)	*p*-Value	Odds Ratio (95%-CI)
Age (mean ± SD, years)	50.1 ± 10.2	50.3 ± 11.0	49.6 ± 7.9	0.721	1.01 (0.96–1.06)
Body mass index (mean ± SD, kg/m^2^)	23.5 ± 3.0	23.5 ± 2.9	23.4 ± 3.2	0.774	1.02 (0.88–1.88)
Underlying disease				NA ^†^	NA
Hypertension	10 (12.2)	9 (15.3)	1 (4.3)		
Hyperlipidemia	10 (12.2)	9 (15.3)	1 (4.3)		
Diabetes mellitus	8 (9.8)	6 (10.2)	2 (8.7)		
Arrhythmia	4 (4.9)	4 (6.8)	0 (0.0)		
Iron deficiency anemia	2 (2.4)	2 (3.4)	0 (0.0)		
Rheumatic disease	3 (3.7)	1 (1.7)	2 (8.7)		
Retinitis pigmentosa	2 (2.4)	1 (1.7)	1 (4.3)		
Bronchiectasis	1 (1.2)	1 (1.7)	0 (0.0)		
Fatty liver	1 (1.2)	1 (1.7)	0 (0.0)		
Polymyositis	1 (1.2)	1 (1.7)	0 (0.0)		
Hypothyroidism	1 (1.2)	0 (0.0)	1 (4.3)		
Systemic lupus erythematosus	1 (1.2)	0 (0.0)	1 (4.3)		
Myocardial infarction	1 (1.2)	0 (0.0)	1 (4.3)		
Bilaterality	5 (6.1)	3 (5.1)	2 (8.7)	0.523	0.56 (0.09–3.38)
Type of breast surgery (n, %)				0.145	0.41 (0.75–1.48)
Breast-conserving surgery	55 (67.1)	38 (64.4)	17 (73.9)		
Mastectomy	27 (32.9)	21 (35.6)	6 (26.1)		
Type of axillary surgery (n, %)				0.197	1.12 (0.85–2.07)
Sentinel lymph node biopsy	64 (78.0)	45 (76.3)	19 (82.6)		
Axillary lymph node dissection	18 (22.0)	14 (23.7)	4 (17.4)		
Breast reconstruction (n, %)	16 (19.5)	14 (23.7)	2 (8.7)	0.147	3.27 (0.69–15.5)
Adjuvant radiotherapy for breast cancer (n, %)	68 (82.9)	46 (78.0)	22 (95.7)	0.090	0.16 (0.02–1.31)
Period between neoadjuvant chemotherapy and operation (mean ± SD, days)	39.8 ± 20.1	43.4 ± 21.9	30.8 ± 10.4	0.058	1.05 (0.96–1.10)

* Immune-related adverse event; ^†^ Not available.

**Table 2:** Disease characteristics of patients who received pembrolizumab-based neoadjuvant chemotherapy (NAC).

Variables	Total (n = 82)	irAE * (n = 59)	No irAE (n = 23)	*p*-Value	
Type of tumor (n, %)				0.783	1.10 (0.79–2.68)
Invasive ductal carcinoma	78 (95.1)	56 (94.9)	22 (95.7)		
Invasive lobular carcinoma	4 (4.9)	3 (5.1)	1 (4.3)		
Clinical tumor size (mean ± SD, cm)	4.0 ± 2.3	4.1 ± 2.4	3.8 ± 2.0	0.643	1.06 (0.84–1.34)
Clinical T stage				0.783	NE ^‡^
T0 ^†^	2 (2.4)	2 (3.4)	0 (0.0)		
T1	3 (3.7)	1 (1.7)	2 (8.7)		
T2	58 (70.7)	42 (71.2)	16 (69.6)		
T3	16 (19.5)	12 (20.3)	4 (17.4)		
T4	3 (3.7)	2 (3.4)	1 (4.3)		
Clinical N stage				0.690	NE
N0	30 (36.6)	22 (37.3)	8 (34.8)		
N1	27 (32.9)	19 (32.2)	8 (34.8)		
N2	14 (17.1)	11 (18.6)	3 (13)		
N3	11 (13.4)	7 (11.9)	4 (17.4)		
Clinical stage				0.783	NE
IIA	26 (31.7)	19 (32.2)	7 (30.4)		
IIB	26 (31.7)	18 (30.5)	8 (34.8)		
IIIA	18 (22.0)	14 (23.7)	4 (17.4)		
IIIB	1 (1.2)	1 (1.7)	0 (0.0)		
IIIC	11 (13.4)	7 (11.9)	4 (17.4)		
Pathologic complete response (n, %)	44 (53.7)	29 (49.2)	15 (65.2)	0.184	0.52 (0.20–1.35)
No tumor	39 (47.6)	25 (42.4)	14 (60.9)		
Ductal carcinoma only	5 (6.1)	4 (6.8)	1 (4.3)		
Pathologic size of invasive carcinoma after NAC (mean ± SD, cm)	1.1 ± 2.8	1.3 ± 3.2	0.8 ± 1.7	0.660	1.15 (0.97–3.10)
No. of metastatic lymph nodes (mean ± SD)	0.9 ± 3.6	0.7 ± 2.6	1.0 ± 4.6	0.311	0.92 (0.79–1.08)
No. of removed lymph nodes (mean ± SD)	7.7 ± 6.9	7.8 ± 4.9	7.3 ± 8.2	0.527	1.02 (0.95–1.10)
Pathologic T stage				0.878	NE
T0	39 (47.6)	25 (42.4)	14 (60.9)		
Tis	5 (6.1)	4 (6.8)	1 (4.3)		
T1	24 (29.3)	19 (32.2)	5 (21.7)		
T2	8 (9.8)	7 (11.9)	1 (4.3)		
T3	4 (4.9)	2 (3.4)	2 (8.7)		
T4	2 (2.4)	2 (3.4)	0 (0.0)		
Pathologic N stage				0.926	NE
N0	73 (89)	53 (89.8)	20 (87)		
N1	4 (4.9)	2 (3.4)	2 (8.7)		
N2	2 (2.4)	1 (1.7)	1 (4.3)		
N3	3 (3.7)	2 (3.4)	1 (4.3)		

* irAE; immune-related adverse event; ^†^ an occult breast cancer case and a recurrent case involving an axillary lymph node; ^‡^ Not estimable (Odds ratios could not be estimated due to sparse data and quasi-complete separation in logistic regression models).

**Table 3:** Immune-related adverse events (irAEs) * related to pembrolizumab in patients who received neoadjuvant chemotherapy.

Organ System	irAE	N (%)	Organ System	irAE	N (%)
Systemic system	Myalgia	33 (40.2)	Gastrointestinal system	Diarrhea	12 (14.6)
	Fever	4 (4.9)		Constipation	9 (11)
Dermatologic system	Skin rash/dermatitis	31 (37.8)		Nausea/vomiting	7 (8.5)
	Soft tissue swelling/cellulitis	1 (1.2)		Dyspepsia	5 (6.1)
	Hyperpigmentation	1 (1.2)		Anal fissure	1 (1.2)
	Herpes zoster infection	1 (1.2)	Respiratory system	Dyspnea on exertion	5 (6.1)
Central nervous system	Peripheral neuropathy	18 (22.0)		Pneumonitis	1 (1.2)
	Headache	1 (1.2)		Pneumonia	1 (1.2)
Musculoskeletal system	Arthralgia	2 (2.4)		Ground-glass opacity	1 (1.2)
	Bone pain	1 (1.2)		NTM infection	1 (1.2)
Endocrine system	Hypothyroidism	12 (14.6)	Ocular system	Uveitis	2 (2.4)
	Hyperthyroidism	4 (4.9)	Hematologic system	Elevation of transaminase	1 (1.2)
	Adrenal insufficiency	1 (1.2)		Anemia	1 (1.2)

* One patient may have multiple irAEs.

**Table 4:** Cases of surgical delay for more than 8 weeks due to immune-related adverse events (irAEs) in patients with triple-negative breast cancer after neoadjuvant chemotherapy.

Patient No.	Age (Years)	Period Between End of NAC and Operation (Days)	irAE	Grade Based on CTCAE v5.0	Abnormal Findings
Patient #33	63	67	Fever, general weakness, mucositis, anemia	Grade 3 (mucositis), grade 3 (anemia)	Hemoglobin 7.0 g/dL, poor oral intake (at cycle #3) → Stop NAC after cycle #5
Patient #35	42	60	General weakness		Poor oral intake (at cycle #8)
Patient #38	35	57	Hypothyroidism	Grade 2	TSH 77.80 mIU/L (at cycle #7) → TSH 0.94 (at 7 weeks after completion of NAC)
Patient #39	48	61	Hypothyroidism	Grade 2	TSH 112.30 mIU/L (at cycle #8) → TSH 3.859 (at 8 weeks after completion of NAC)
Patient #55	51	80	Increased transaminase (AST/ALT)	Grade 3	AST/ALT 841/502 U/L (at cycle #1) → Stop NAC after cycle #1
Patient #78	51	62	Hypothyroidism	Grade 2	TSH 57.88 mIU/L (at baseline) → TSH 68.204 (at 2 weeks after completion of NAC) → TSH 1.807 (at 7 weeks after completion of NAC)

AST, aspartate transaminase; ALT, alanine transferase; TSH, thyroid-stimulating hormone.

## Data Availability

The datasets generated and analyzed during the current study are not publicly available. However, they are available from the corresponding author upon reasonable request.

## References

[1] Starnes C.O. (1992). Coley’s toxins in perspective. Nature.

[2] Bickels J., Kollender Y., Merinsky O., Meller I. (2002). Coley’s toxin: Historical perspective. Isr. Med. Assoc. J..

[3] Waldman A.D., Fritz J.M., Lenardo M.J. (2020). A guide to cancer immunotherapy: From T cell basic science to clinical practice. Nat. Rev. Immunol..

[4] Zhang Y., Zhang Z. (2020). The history and advances in cancer immunotherapy: Understanding the characteristics of tumor-infiltrating immune cells and their therapeutic implications. Cell Mol. Immunol..

[5] Li C.H., Karantza V., Aktan G., Lala M. (2019). Current treatment landscape for patients with locally recurrent inoperable or metastatic triple-negative breast cancer: A systematic literature review. Breast Cancer Res..

[6] Nanda R., Chow L.Q., Dees E.C., Berger R., Gupta S., Geva R., Pusztai L., Pathiraja K., Aktan G., Cheng J.D. (2016). Pembrolizumab in Patients With Advanced Triple-Negative Breast Cancer: Phase Ib KEYNOTE-012 Study. J. Clin. Oncol..

[7] Adams S., Loi S., Toppmeyer D., Cescon D.W., De Laurentiis M., Nanda R., Winer E.P., Mukai H., Tamura K., Armstrong A. (2019). Pembrolizumab monotherapy for previously untreated, PD-L1-positive, metastatic triple-negative breast cancer: Cohort B of the phase II KEYNOTE-086 study. Ann. Oncol..

[8] Schmid P., Cortes J., Pusztai L., McArthur H., Kümmel S., Bergh J., Denkert C., Park Y.H., Hui R., Harbeck N. (2020). Pembrolizumab for Early Triple-Negative Breast Cancer. N. Engl. J. Med..

[9] Schmid P., Cortes J., Dent R., Pusztai L., McArthur H., Kümmel S., Bergh J., Denkert C., Park Y.H., Hui R. (2022). Event-free Survival with Pembrolizumab in Early Triple-Negative Breast Cancer. N. Engl. J. Med..

[10] Kwok G., Yau T.C., Chiu J.W., Tse E., Kwong Y.L. (2016). Pembrolizumab (Keytruda). Hum. Vaccin. Immunother..

[11] Wang P.F., Chen Y., Song S.Y., Wang T.J., Ji W.J., Li S.W., Liu N., Yan C.X. (2017). Immune-Related Adverse Events Associated with Anti-PD-1/PD-L1 Treatment for Malignancies: A Meta-Analysis. Front. Pharmacol..

[12] Zhao Q., Zhang J., Xu L., Yang H., Liang N., Zhang L., Zhang F., Zhang X. (2021). Safety and Efficacy of the Rechallenge of Immune Checkpoint Inhibitors After Immune-Related Adverse Events in Patients With Cancer: A Systemic Review and Meta-Analysis. Front. Immunol..

[13] Lei C., Kong X., Li Y., Yang H., Zhang K., Wang Z., Chang H., Xuan L. (2024). PD-1/PD-L1 Inhibitor—Related Adverse Events and Their Management in Breast Cancer. J. Cancer.

[14] Zhang M., Song J., Yang H., Jin F., Zheng A. (2022). Efficacy and safety of PD-1/PD-L1 inhibitors in triple-negative breast cancer: A systematic review and meta-analysis. Acta Oncol..

[15] Reck M., Remon J., Hellmann M.D. (2022). First-Line Immunotherapy for Non-Small-Cell Lung Cancer. J. Clin. Oncol..

[16] Motzer R.J., Porta C., Eto M., Powles T., Grünwald V., Hutson T.E., Alekseev B., Rha S.Y., Merchan J., Goh J.C. (2024). Lenvatinib Plus Pembrolizumab Versus Sunitinib in First-Line Treatment of Advanced Renal Cell Carcinoma: Final Prespecified Overall Survival Analysis of CLEAR, a Phase III Study. J. Clin. Oncol..

[17] Motzer R., Alekseev B., Rha S.Y., Porta C., Eto M., Powles T., Grünwald V., Hutson T.E., Kopyltsov E., Méndez-Vidal M.J. (2021). Lenvatinib plus Pembrolizumab or Everolimus for Advanced Renal Cell Carcinoma. N. Engl. J. Med..

[18] U.S. Department of Health and Human Services (2017). Common Terminology Criteria for Adverse Events (CTCAE).

[19] Emens L.A., Adams S., Cimino-Mathews A., Disis M.L., Gatti-Mays M.E., Ho A.Y., Kalinsky K., McArthur H.L., Mittendorf E.A., Nanda R. (2021). Society for Immunotherapy of Cancer (SITC) clinical practice guideline on immunotherapy for the treatment of breast cancer. J. Immunother. Cancer.

[20] Lee J.S., Yost S.E., Yuan Y. (2020). Neoadjuvant Treatment for Triple Negative Breast Cancer: Recent Progresses and Challenges. Cancers.

[21] Li Y., Zhang H., Merkher Y., Chen L., Liu N., Leonov S., Chen Y. (2022). Recent advances in therapeutic strategies for triple-negative breast cancer. J. Hematol. Oncol..

[22] Han H.S., Vikas P., Costa R.L.B., Jahan N., Taye A., Stringer-Reasor E.M. (2023). Early-Stage Triple-Negative Breast Cancer Journey: Beginning, End, and Everything in Between. Am. Soc. Clin. Oncol. Educ. Book.

[23] Pusztai L., Denkert C., O’Shaughnessy J., Cortes J., Dent R., McArthur H., Kümmel S., Bergh J., Park Y.H., Hui R. (2024). Event-free survival by residual cancer burden with pembrolizumab in early-stage TNBC: Exploratory analysis from KEYNOTE-522. Ann. Oncol..

[24] Choi J., Lee S.Y. (2020). Clinical Characteristics and Treatment of Immune-Related Adverse Events of Immune Checkpoint Inhibitors. Immune Netw..

[25] Okwundu N., Grossman D., Hu-Lieskovan S., Grossmann K.F., Swami U. (2021). The dark side of immunotherapy. Ann. Transl. Med..

[26] Vaddepally R., Doddamani R., Sodavarapu S., Madam N.R., Katkar R., Kutadi A.P., Mathew N., Garje R., Chandra A.B. (2022). Review of Immune-Related Adverse Events (irAEs) in Non-Small-Cell Lung Cancer (NSCLC)-Their Incidence, Management, Multiorgan irAEs, and Rechallenge. Biomedicines.

[27] Zhao Z., Wang X., Qu J., Zuo W., Tang Y., Zhu H., Chen X. (2021). Immune-Related Adverse Events Associated With Outcomes in Patients With NSCLC Treated With Anti-PD-1 Inhibitors: A Systematic Review and Meta-Analysis. Front. Oncol..

[28] Shankar B., Zhang J., Naqash A.R., Forde P.M., Feliciano J.L., Marrone K.A., Ettinger D.S., Hann C.L., Brahmer J.R., Ricciuti B. (2020). Multisystem Immune-Related Adverse Events Associated With Immune Checkpoint Inhibitors for Treatment of Non-Small Cell Lung Cancer. JAMA Oncol..

[29] Hata H., Matsumura C., Chisaki Y., Nishioka K., Tokuda M., Miyagi K., Suizu T., Yano Y. (2022). A Retrospective Cohort Study of Multiple Immune-Related Adverse Events and Clinical Outcomes Among Patients With Cancer Receiving Immune Checkpoint Inhibitors. Cancer Control.

[30] Washino S., Shirotake S., Takeshita H., Inoue M., Miura Y., Hyodo Y., Kagawa M., Izumi K., Oyama M., Kawakami S. (2023). Association between immune-related adverse events and survival in patients with renal cell carcinoma treated with nivolumab plus ipilimumab: Immortal time bias-corrected analysis. Int. J. Clin. Oncol..

[31] Ikeda T., Ishihara H., Nemoto Y., Tachibana H., Fukuda H., Yoshida K., Takagi T., Iizuka J., Hashimoto Y., Ishida H. (2021). Prognostic impact of immune-related adverse events in metastatic renal cell carcinoma treated with nivolumab plus ipilimumab. Urol. Oncol..

[32] Nukaya T., Takahara K., Yoshizawa A., Saruta M., Yano Y., Ohno T., Uchimoto T., Fukuokaya W., Adachi T., Yamazaki S. (2024). Prognostic Impact of Immune-Related Adverse Events as First-Line Therapy for Metastatic Renal Cell Carcinoma Treated with Nivolumab Plus Ipilimumab: A Multicenter Retrospective Study. Clin. Genitourin. Cancer.

[33] Hak A.E., Pols H.A., Visser T.J., Drexhage H.A., Hofman A., Witteman J.C. (2000). Subclinical hypothyroidism is an independent risk factor for atherosclerosis and myocardial infarction in elderly women: The Rotterdam Study. Ann. Intern. Med..

[34] Loeliger E.A., Van Der Esch B., Mattern M.J., Hemker H.C. (1964). The Biological Disappearance Rate of Prothrombin, Factors VII, IX and X from Plasma in Hypothyroidism, Hyperthyroidism, and During Fever. Thromb. Diath. Haemorrh..

[35] Thoyyib M., Garg S., Gupta N., Aggarwal S., Pandit S. (2018). Study on Coagulation Factor VIII and Fibrinogen Levels in Patients with Thyroid Disorders. Indian J. Endocrinol. Metab..

[36] Fredlund B.O., Olsson S.B. (1983). Long QT interval and ventricular tachycardia of “torsade de pointe” type in hypothyroidism. Acta Med. Scand..

[37] Udovcic M., Pena R.H., Patham B., Tabatabai L., Kansara A. (2017). Hypothyroidism and the Heart. Methodist. Debakey Cardiovasc. J..

[38] Klein I., Ojamaa K. (2001). Thyroid hormone and the cardiovascular system. N. Engl. J. Med..

[39] Klein I., Danzi S. (2007). Thyroid disease and the heart. Circulation.

[40] Lipp H.P., Kahaly G.J. (2021). Administration and Pharmacokinetics of Levothyroxine. 70 Years of Levothyroxine.

[41] Colucci P., Yue C.S., Ducharme M., Benvenga S. (2013). A Review of the Pharmacokinetics of Levothyroxine for the Treatment of Hypothyroidism. Eur. Endocrinol..

[42] Martin C.A., Nazareth J., Jarkhi A., Pan D., Das M., Logan N., Scott S., Bryant L., Abeywickrama N., Adeoye O. (2023). Ethnic differences in cellular and humoral immune responses to SARS-CoV-2 vaccination in UK healthcare workers: A cross-sectional analysis. EClinicalMedicine.

[43] Liston A., Humblet-Baron S., Duffy D., Goris A. (2021). Human immune diversity: From evolution to modernity. Nat. Immunol..

